# Application of a Clinical Workflow May Lead to Increased Diagnostic Precision in Hereditary Spastic Paraplegias and Cerebellar Ataxias: A Single Center Experience

**DOI:** 10.3390/brainsci11020246

**Published:** 2021-02-16

**Authors:** Vittorio Riso, Salvatore Rossi, Tommaso F. Nicoletti, Alessandra Tessa, Lorena Travaglini, Ginevra Zanni, Chiara Aiello, Alessia Perna, Melissa Barghigiani, Maria Grazia Pomponi, Filippo M. Santorelli, Gabriella Silvestri

**Affiliations:** 1 UOC Neurologia, Fondazione Policlinico Universitario ‘A. Gemelli’ IRCCS, 00168 Rome, Italy; salvatorerossi309@gmail.com (S.R.); tommasof.nicoletti@gmail.com (T.F.N.); alessia1perna@gmail.com (A.P.); gabriella.silvestri@unicatt.it (G.S.); 2 Department of Neurosciences, Università Cattolica del Sacro Cuore, 00168 Rome, Italy; 3 Molecular Medicine Unit, IRCCS Fondazione Stella Maris, 56018 Pisa, Italy; aletessa@gmail.com (A.T.); mely.b91@hotmail.com (M.B.); filippo3364@gmail.com (F.M.S.); 4 Unit of Muscular and Neurodegenerative Diseases, Department of Neurosciences, Bambino Gesù Children’s Hospital, 00165 Rome, Italy; lorena.travaglini@opbg.net (L.T.); ginevra.zanni@opbg.net (G.Z.); chiara.aiello@opbg.net (C.A.); 5 Genetics and Rare Diseases Research Division, Bambino Gesù Children’s Hospital, 00165 Rome, Italy; 6 UOC Genetica Medica, Fondazione Policlinico Universitario “A.Gemelli” IRCCS, 00168 Rome, Italy; mariagrazia.pomponi@policlinicogemelli.it

**Keywords:** NGS, HSP, SCA, hereditary spastic paraplegia, ataxia, neurogenetics

## Abstract

The molecular characterization of Hereditary Spastic Paraplegias (HSP) and inherited cerebellar ataxias (CA) is challenged by their clinical and molecular heterogeneity. The recent application of Next Generation Sequencing (NGS) technologies is increasing the diagnostic rate, which can be influenced by patients’ selection. To assess if a clinical diagnosis of CA/HSP received in a third-level reference center might impact the molecular diagnostic yield, we retrospectively evaluated the molecular diagnostic rate reached in our center on 192 unrelated families (90 HSP and 102 CA) (i) before NGS and (ii) with the use of NGS gene panels. Overall, 46.3% of families received a genetic diagnosis by first-tier individual gene screening: 43.3% HSP and 50% spinocerebellar ataxias (SCA). The diagnostic rate was 56.7% in AD-HSP, 55.5% in AR-HSP, and 21.2% in sporadic HSP. On the other hand, 75% AD-, 52% AR- and 33% sporadic CA were diagnosed. So far, 32 patients (24 CA and 8 HSP) were further assessed by NGS gene panels, and 34.4% were diagnosed, including 29.2% CA and 50% HSP patients. Eleven novel gene variants classified as (likely) pathogenic were identified. Our results support the role of experienced clinicians in the diagnostic assessment and the clinical research of CA and HSP even in the next generation era.

## 1. Introduction

Until a few years ago, the diagnostic route of Hereditary Spastic Paraplegias (HSPs) and cerebellar ataxias (CA) was time-consuming, cost expensive and challenging in many cases because of their clinical and genetical heterogeneity.

So far, at least 64 genes (the SPastic Gait/Gene or SPG genes) have been characterized, and further HSP loci have been mapped [1,2,3,4,5,6,7]. Most autosomal dominant (AD)-HSPs are pure, and SPG4, due to mutations in *SPAST*, is the most common genetic variant [1,2,3] Mutations in *ATL1* (SPG3A), *REEP1* (SPG31), and *KIF5A* (SPG10) are also relatively frequent; when combined with SPG4, these forms account for about 50–60% of AD-HSP families [8,9,10].

Autosomal recessive (AR)-HSPs are mostly complicated and more heterogeneous than AD-HSP, with at least 50 SPG loci (and 45 causal genes) so far identified [3]. SPG11, due to mutations in the spatacsin gene, is the most frequent form, including about 20% of all AR-HSP and up to 45% of AR-HSP with a thin corpus callosum (TCC) [11,12]. Additional genes, including *CYP7B1* (SPG5), paraplegin (SPG7), and *ZFYVE26* (SPG15) [7,8,9,10], totally account for another 20% of AR-HSP. While X-linked HSPs are extremely rare [1,2,3], it is noteworthy that specific HSP genes have been recently associated with both AD and AR forms, depending on the effect of the mutations [13,14,15,16,17,18,19].

Inherited degenerative cerebellar ataxias (CA or spinocerebellar ataxias, SCAs) include over 100 related genes and all possible modes of transmission [20]. Both the autosomal dominant (ADCAs) and the autosomal recessive variants (SCAR/ARCAs) are numbered in the chronological order in which loci were identified [20]. ADCA usually have an adult onset, and they are most frequently caused by pathological expansions of CAG repeats in seven genes, all encoding for polyglutamine (polyQ) tracts. They globally account for 40–60% of ADCA cases, whereas about 3–5% of dominant forms are caused by either non-coding repeat expansions or conventional mutations in further genes [20].

ARCAs usually have an infantile or juvenile onset; their genetic diagnosis is more challenging because of a possible clinical overlap with other rare neurogenetic disorders [21,22]. Sporadic HSP and SCA can also occur, and a genetic etiology is identified in a minority of these patients.

The first significant progress in the genetic and pathogenic characterization of SCAs and HSPs occurred with the identification of the poly-Q repeat expansions as a frequent cause of ADCAs, of pathological GAA expansion in the *FXN* gene associated with Friedreich ataxia, and of mutations or major rearrangements located in the SPAST gene in association with pure HSP (SPG4).

Molecular screening studies on large cohorts of SCA/HSP patients indicated that such genetic etiologies could account for no more than 30–50% of the familial cases, with a distinct prevalence in the various populations. In the following years, the identification of many novel SCA/HSP genes confirmed their wide genetic heterogeneity. Thus, the use of individual gene screening required constant updates of the applied diagnostic flowcharts, and eventually became time-consuming, expensive, and applicable only by a few research centers.

The development of Next Generation Sequencing (NGS) technologies has marked a turning point in the diagnosis of HSPs and ARCA/SCAs. NGS analysis by targeted resequencing multigene panels (TRPs) represents the most cost-effective approach, allowing high deep coverage of the coding exons of a variable number of known disease-related genes at once. Additional high-throughput NGS methods are whole-exome sequencing (WES), covering the full set of DNA encoding sequences, and whole-genome sequencing (WGS), representing the most expensive, all-inclusive technique [23].

The use of NGS panels has improved (i) the rate and timing of genetic characterization in HSP and SCA patients and (ii) the definition of the relative prevalence and phenotypic spectrum of individual forms [24,25], also documenting a continuum spastic/ataxic clinical spectrum related to functionally clustered genes [26]. WES or WGS represent an additional turning point allowing the identification of novel SCA/HSP genes, a prerequisite to shed further light on related pathogenic mechanisms and future treatments [27,28].

The diagnostic rate for SCA and HSP varies between different studies, and this may be influenced not only by the type of NGS approach applied, but also by the criteria adopted to select patients in third-level reference centers. Here we report the results of a retrospective study conducted on a large cohort of HSP/SCA patients referred to us for diagnosis and follow-up over a 14-year period (from 2005 to 2019). Our aims were to estimate the diagnostic yield reached in this HSP and SCA cohorts i) before NGS ii) by NGS gene panels.

## 2. Materials and Methods

This retrospective study was carried out in compliance with the Helsinki Declaration and the Good Clinical Practice; all patients gave a written informed consent, approved by our ethics committee, authorizing the use of diagnostic data for clinical research purposes about their disease.

**Figure 1:**
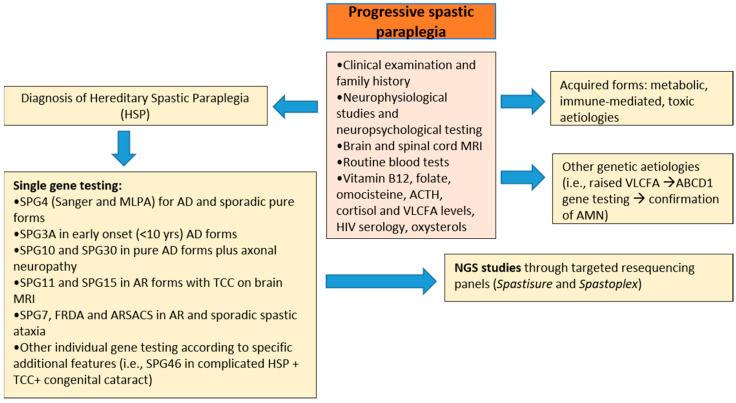
Diagnostic flowchart applied to patients with suspected degenerative progressive spastic paraplegia (HSP) Abbreviations: NGS, Next Generation Sequencing; VLCFA, Very Long Chain Fatty Acids; AD, autosomal dominant; AR, autosomal recessive; TCC, thin corpus callosum; FRDA, Freidreich’s Ataxia; ARSACS, Autosomal recessive spastic ataxia of Charlevoix-Saguenay; MLPA, Multiplex Ligation Probe Amplification; HIV, Human Immunodeficiency Virus; AMN, adrenomyeloneuropathy.

**Figure 2:**
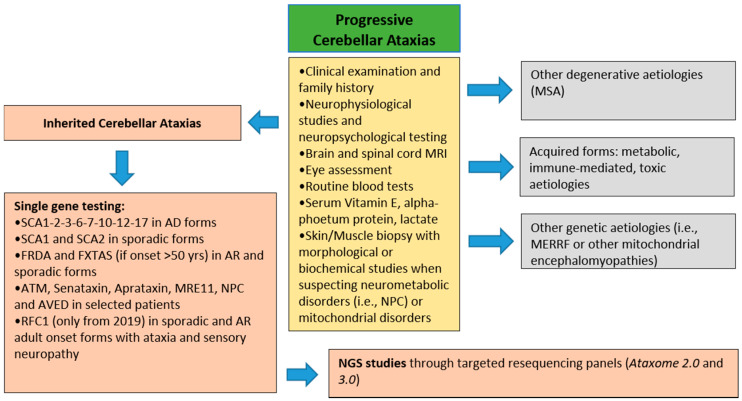
Diagnostic flowchart applied at the suspected degenerative cerebellar ataxia patients. Abbreviations: SCA, SpinoCerebellar Ataxia; FRDA, Friedreich’s Ataxia; FXTAS, Fragile X-associated tremor/ataxia syndrome; AD, autosomal dominant; AR, autosomal recessive; ATM, ataxia-telangiectasia mutated; NPC, Niemann-Pick disease type C; AVED, Ataxia with vitamin E deficiency; RFC1, replication factor complex 1; NGS, Next-Generation Sequencing.

It involved a total of 230 consecutive adult patients from 192 unrelated families: 124 SCA (mean age 58.4 years, range 19–86 years) from 102 families and 106 HSPs (mean age 53.4 years, range 18–86 years) from 90 families. All of them were clinically evaluated and diagnosed at our third-level expert center for diagnosis and follow-up for HSPs and cerebellar ataxias in the Latium Region over a 14-year period (from January 2005 until December 2019). Prior to this study, other causes of toxic, immune-mediated, inflammatory, and metabolic ataxias and spastic paraplegia had been ruled out by appropriate clinical tests (Figure 1; Figure 2).

The SCA cohort included 32 ADCA families (31.4%), 31 ARCA families (30.4%), and 39 sporadic (38.2%). MSA-C patients were not included. Among HSP families, 30/90 (33.3%) were AD, 27 (30%) were AR, and 33 (36.7%) sporadic (Table 1).

The diagnostic protocol for cerebellar ataxias and HSP, illustrated in Figure 1; Figure 2, respectively, included brain and spine MRI, EEG, EMG and nerve conduction studies, motor- and sensory-evoked potentials, ophthalmological, audiological, and neuropsychological evaluation. Routine blood tests also included vitamin B12, homocysteine and folate plasma levels; other tests performed were serum vitamin E, lactate and alpha-foetum protein levels, plasma ceruloplasmin, and copper and iron determinations. ACTH, cortisol, and plasma very long-chain fatty acid (VLCFA) levels were also assessed in all males with spastic ataxia with onset <40 years of age. In selected cases, skin or muscle biopsy was performed when Niemann Pick type C or mitochondrial diseases were suspected, respectively, and lysosomal HEXA and B activities in leukocytes to rule out Sandhoff disease. Immunoblotting studies of ATM, senataxin, aprataxin, and MRE11 proteins were done in all ataxic cases showing clinical oculomotor apraxia and/or raised serum alpha-fetoprotein levels.

Leukocyte total DNA was isolated from blood samples after informed consent and processed for primary diagnostic purposes at the Medical Genetics Laboratory of Fondazione Policlinico A. Gemelli (Rome).

For HSP patients, first-tier individual gene screening (Figure 1) included analysis of *SPAST* (SPG4) gene by direct Sanger sequencing and Multiplex Ligation Probe Amplification (MLPA) in all AD or sporadic pure forms, SPG3A in all AD cases with onset <10 years, and search for variants in SPG10 and SPG30 either in pure AD forms or those complicated by axonal neuropathy. Direct capillary sequencing of the SPG11 and SPG15 genes was done in all AR-HSP with TCC on brain MRI.

In cases with sporadic or documented AR inheritance of spastic ataxia, we sought pathological GAA expansion of the first intron of the *FXN* gene and direct sequencing of the *SACS* and *SPG7* genes.

In the first-tier screening (Figure 2), all AD SCA patients underwent screening for expanded repeats at the AD-SCA1-2-3-6-7-10-12-17. ARCA and sporadic SCA were screened for expanded repeats at FRDA and FTXAS (if onset >50 years) loci; sporadic SCA performed SCA1 and SCA2 testing as well (as these are the most frequent AD-SCA in Italy). DNA samples that tested negative were sent for further diagnostic testing by Sanger sequencing of individual SCA or HSP genes, according to the suspicion emerging from results of the diagnostic assessment (i.e., *TTPA* in the case of very low or undetectable serum vitamin E, *ATM* in the case of raised alfa-fetoprotein levels, direct sequencing of the SCA28/*AFG3L2* gene in patients with autosomal dominant spastic ataxia) either to the Molecular Medicine Laboratory, IRCCS Stella Maris, Pisa, or the Laboratory of Molecular medicine Ospedale Pediatrico Bambino Gesù, Rome. More recently, late-onset sporadic or ARCA patients with sensory ataxia were also tested for biallelic AAGGG expansion in *RFC1* associated with CANVAS [29].

At the time of this writing, 32 patients completed NGS investigations using targeted multigene panels (TRPs) designed for ataxia (ATAXOME 2.0 and 3.0, which contained 273 and 285 genes, respectively), HSP (Spastoplex with 72 genes and Spastisure with 118), and leukodystrophies. DNA samples were analyzed either at the Fondazione Stella Maris IRCCS (28 pts) or at the Ospedale Pediatrico Bambino Gesù (4 pts). Details about NGS methodology, accuracy of the specific TRP methodology, and customized bioinformatics pipelines have been previously described [25,30,31,32]. Variants were classified as pathogenic, likely pathogenic, of uncertain significance (VUS), likely benign or benign according to the criteria established by the ACMG standards and guidelines [33]. All variants were further validated by capillary Sanger sequencing, and family segregation studies were carried out if DNA from close relatives was available.

## 3. Results

Eighty-nine out of 192 HSP/SCA families (46.3%) received a genetic diagnosis at the first-tier screening.

**Figure 3:**
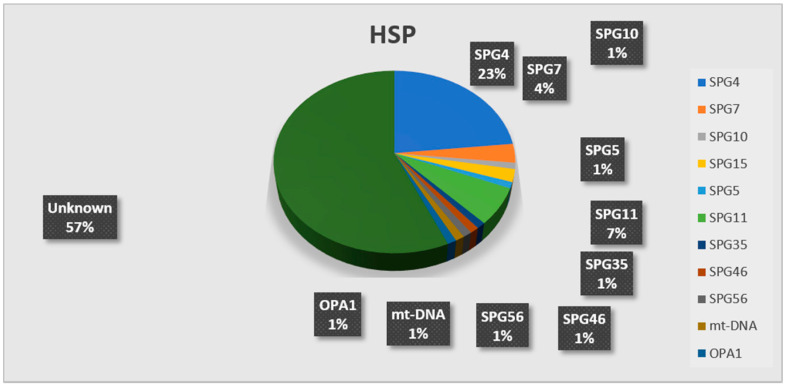
The pie-chart displays the results of individual gene screening in the HSP cohort, with an overall diagnostic yield of 43%. In black boxes, the relative percentages for each molecular diagnosis are reported; SPG4 was the most common HSP form, followed by SPG11 and SPG7.

In total, 39/90 (43.3%) HSP probands were genetically characterized (Table 1 and Figure 3); the diagnostic rate was 56.7% in AD-HSP (17/30 families), and SPG4 was the most frequent form (16/17), with a novel SPG10 pathogenic variant found in one family [34]. Regarding AR-HSP, a genetic diagnosis was made in 15/27 families (55.5%): in the SPG11 gene, mutations were the prevalent genetic etiology (six families, 42.8%), followed by SPG15 and SPG7 (six families each), SPG5, SPG35, SPG46, and SPG56 [35] (one patient each). A 35-year-old female with severe spastic paraplegia and optic atrophy, included among AR cases as one deceased sister was similarly affected, carried a pathogenic variant affecting a conserved position in the mtDNA-encoded gene *MT-ND3* gene. Her muscle biopsy showed a biochemical complex I deficiency, and no ragged red fibers (RRFs). Seven patients out of 33 sporadic HSPs (21.2%) received a genetic diagnosis: five harbored mutations in *SPAST*, one case had biallelic variants in *SPG7*, and a young woman manifesting with HSP + early onset optic atrophy harbored a nonsense variant in *OPA1*.

**Figure 4:**
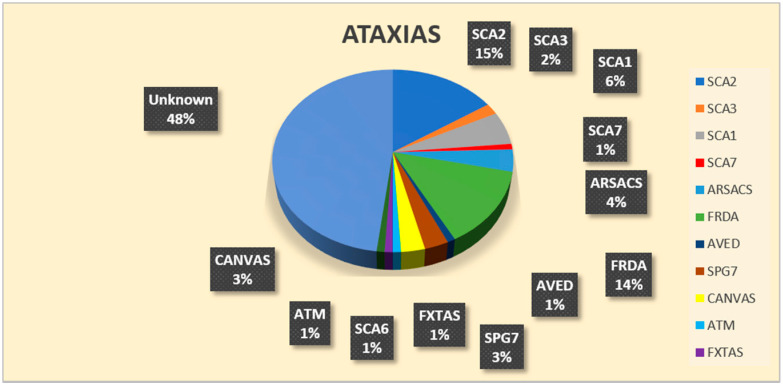
Pie-chart showing the results of individual gene screening on the group degenerative ataxias. A molecular diagnosis was assessed in 52% of the whole cohort. In black boxes the relative percentages for each molecular diagnosis are reported; SCA2 and Friedreich’s ataxia were the most common genetic subtypes.

Regarding SCAs, a genetic diagnosis was achieved in 53/102 families (52%) (Table 1 and Figure 4). Specifically, 24/32 AD forms (75%) were polyQ SCA, with SCA2 being the most frequent (15 families), followed by SCA1 (five families), SCA3 (two families), SCA6, and SCA7 (one family each). One SCA3 patient and the SCA7 patient were not of Italian ancestry. Among the 31 ARCA families, 16 (51.6%) received a genetic diagnosis. In this case, FRDA was the most frequent etiology (10 families), followed by *SACS* (3 families), and one case each received a molecular diagnosis of ATM, AVED, and CANVAS. The AVED family was of Moroccan origin. Finally, 13/39 sporadic patients with ataxia (33.3%) received a genetic diagnosis as follows: four with homozygous intron 1 expansion in FXN; three biallelic variants in SPG7, 2 CANVAS, 1 SACS, 1 SCA1, 1 SCA2; and one FXTAS.

### NGS Studies

**Figure 5:**
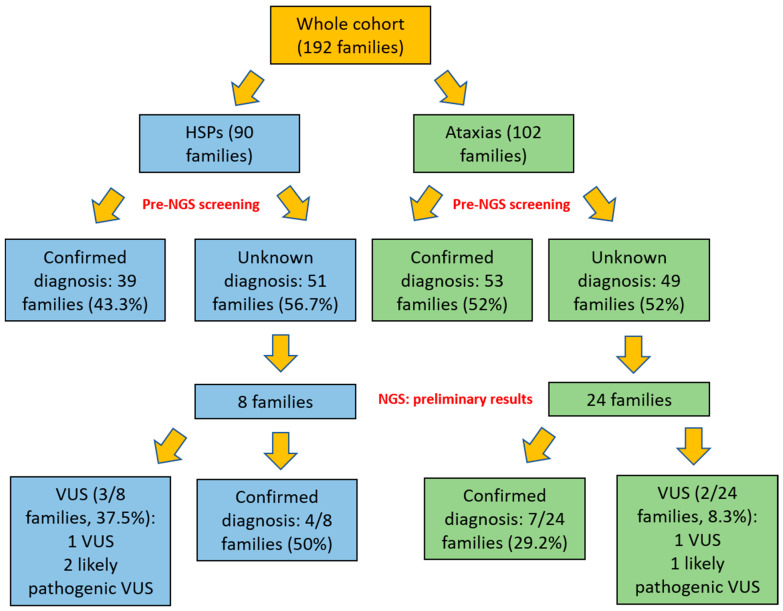
Schematic representation of the results of the diagnostic route in our cohort of SCA and HSP cases.

Until now, TRP-NGS based molecular screening was completed in 32/103 of our undiagnosed families, including 24 with cerebellar or spastic ataxia, and 8 with pure or complicated (Figure 5). Overall, 11/32 cases (34.4%) had a confirmatory molecular diagnosis: 7/24 (29.2%) had cerebellar ataxia, and 4/8 (50%) presented HSP. Eleven of the identified variants classified as likely pathogenic were novel.

In addition, two further patients with ataxia and three with HSP showed VUS in six genes (Table 2) five of which with a possible pathogenic significance, deserving further investigations to assess a disease-related role

Table 2 summarizes clinical and molecular findings of these 16 patients. Six patients (#1, #2, #5, #11, #12, #13) have been already described [30,32,36]. Clinico-diagnostic features are reported in detail in a supplementary file.

## 4. Discussion

This retrospective study aimed to assess the value of our clinical-diagnostic assessment in patients with suspected HSP or SCA either before or, although on limited data, following the advent of targeted NGS panels in the genetic characterization of these two neurodegenerative forms.

In our first-tier screening based on individual gene testing, 46% of 205 families received a diagnosis, specifically 43.3% of HSP and 50% of the cerebellar ataxias. SPG4 form was the most frequent among AD-HSP, and the SPG11 form among AR-HSP. The diagnostic rate was lower, but relevant (21.21%) also in sporadic HSPs. Among cerebellar ataxias, ADCA showed a higher diagnostic rate (64.5%), mostly due to polyQ AD-SCA. Fifty-two percent of ARCA was also genetically diagnosed, and FRDA was the most frequent form, followed by ARSACS. Sporadic SCAs showed a diagnostic rate of 33.3%; FRDA and SPG7 were equally prevalent in this subcohort, followed by ARSACS and CANVAS.

These results support the consistency and accuracy of our diagnostic protocol specifically designed for SCAs and HSPs. In fact, a recent review indicated that the number of families without a genetic diagnosis after systematic gene testing ranged from 33 to 92% in the ADCA group, 40–46% in the ARCA, 45–67% in the AD-HSP, and 71–82% in the AR-HSP groups [1].

Remarkably, among diagnosed HSP families, we did not include those that manifested spastic paraparesis as a predominant feature, in whom we addressed by our diagnostic protocol other genetic diagnoses, including one family affected by severe MTHFR deficiency [40], and two unrelated males with adrenomyeloneuropathy. On the other hand, we also included three patients who recently tested positive to the biallelic expanded pentanucleotide in *RFC1* associated with CANVAS.

The advent of the “next-generation sequencing era” has positively changed the perspective for clinical research and diagnosis also regarding SCA and HSP [24,25,41,42]; a recent review of the literature of NGS-based studies in ataxias assessed an average diagnostic rate of 17% for TRPs, increasing to 34% when including VUS with a potential pathogenic role. As expected, WES resulted in a higher average diagnostic rate (36%, range 21–46%), again with higher rates (average 53%) if VUS of potential pathogenic significance were also included [25].

Regarding HSP, two distinct NGS panels including over 100 HSP/ataxia genes applied to a very large cohort HSP patients, who obtained a positive diagnostic yield of 29%, with VUS of possible pathogenic significance found in an additional 36% of cases [30].

Comparing these data with our preliminary findings by TRPs, we obtained a good diagnostic rate in ataxias (29%, 7/24 cases), being even higher (50% 4/8) in HSP without including VUS.

Table 3 illustrates diagnostic rates of the more recent and largest NGS studies along with our work.

As expected, studies applying WES/MLPA only to familial HSP or SCA cases reached higher diagnostic yields (50–60%) [8,47], even up to 90% when regarding only AD-HSP [48]. Conversely, similar studies obtained a lower diagnostic rate (33%) in cohorts of previously undiagnosed patients and also including sporadic cases [8].

However, a detailed clinical assessment still plays a significant role, as supported by recent works: (i) a direct high-throughput next-generation approach using Trusight obtained a diagnostic rate of 50% [8], which in fact increased to 70% when applied to well-selected HSP patients negative to previous genetic screenings [8,45]; (ii) a NGS multigene panel-based molecular screening assessed in 50 ataxic patients tested negative for SCA1, -2, -3, -6, and -7, and Friedreich ataxia had an overall detection rate of 18%, being lower (8.3%) in adult-onset forms, higher (40%) in childhood or adolescent-onset progressive disorders, and highest (75%) in those with adolescent-onset and a positive family history [41].

## 5. Conclusions

In conclusion, results from our work would support the role of experienced clinicians in the diagnostic assessment of SCA/HSP patients to reach a conclusive molecular definition, even in “the next generation era”, as they can offer a precious contribution to geneticists both in the patients’ selection and in the critical evaluation of the gene variants prioritized in silico.

## Figures and Tables

**Table 1:** Genetic diagnosis reached at the first-tier screening. Abbreviations: HSP, Hereditary Spastic Paraplegia; CA, Cerebellar Ataxia; AD, autosomal dominant; AR, autosomal recessive; SGT, single gene testing.

**HSP (90 Families)**			
	AD 30/90 (33.3%)	AR 27/90 (30%)	Sporadic 33/90 (36.7%)
diagnosis reached	17/30 (56.7%)	15/27 (55.5%)	7/33 (21.2%)
genetic diagnosis	16 SPG4/30 SPG4 SGT 1 SPG10/1 SPG10 SGT	6 SPG11/8 SPG11 SGT 2 SPG15/2 SPG15 SGT 2 SPG7/5 SPG7 SGT 1 SPG5/1 SPG5 SGT 1 SPG35/1 SPG35 SGT 1 SPG45/1 SPG45 SGT 1 SPG56/4 SPG56 SGT 1 MT-ND3/1 mtDNA sequencing	5 SPG4/33 SPG4 SGT 1 SPG7/6 SPG7 SGT 1 OPA1/1 OPA1 SGT
**CA (102 Families)**			
	AD 32/102 (31.4%)	AR 31/102 (30.4%)	Sporadic 39/102 (38.2%)
diagnosis reached	24/32 (75%)	16/31 (51.6%)	13/39 (33.3%)
genetic diagnosis	15 SCA2/32 SCA2 SGT 5 SCA1/32 SCA1 SGT 2 SCA3/32 SCA3 SGT 1 SCA6/32 SCA6 SGT 1 SCA7/32 SCA7 SGT	10 FRDA/31 FRDA SGT 3 SACS/4 SACS SGT 1 ATM/1 ATM SGT 1 AVED/1 TTPA SGT 1 CANVAS/2 RFC1 SGT	4 FRDA/39 FRDA SGT 3 SPG7/6 SPG7 SGT 2 CANVAS/20 RFC1 SGT 1 SACS/7 SACS SGT 1 SCA1/39 SCA1 SGT 1 SCA2/39 SCA2 SGT 1 FXTAS/6 FMR1 SGT

**Table 2:** Demographic data and clinical/diagnostic findings in HSP and SCA patients successfully assessed by NGS targeted gene panels. Novel variants are reported in bold. AR: autosomal recessive; AD: autosomal dominant; SP: sporadic; TCC: thin corpus callosum; CA: cerebellar atrophy OPCA: Olivo-ponto-cerebellar atrophy; POLR3A: Polymerase RNA III. Related references are reported in brackets. The line in bold divides HSP and ataxias cases.

Patient	Age of Onset	Age at Diagnosis	Family History	Phenotype	Neuroimaging	Gene/Disease	Variant
#1 [30]	25	25	AR	Pure HSP + ny	normal	*CYP721*/SPG5 NM_004820	c.338insT/p.Phe114fs*3 **(Homozygous)**
#2 [30]	15	37	AR	HSP, epilepsy and cognitive delay	TCC and spinal cord atrophy	*KIAA1840*/SPG11 NM_025137	c.2833A > G/p.Arg945Gly + c.128delC/p.S43fs*15
#3	20	37	AR	Pure HSP	normal	*PGN*/SPG7 NM_003119	c.1369C > T/p.R457* + c.1617delC/p.V540Cfs*52
#4	20	35	AR	HSP + mild hyperCK	Mild TCC	*KIAA1840*/SPG11 NM_025137	c.2842-2843insG/p.Val948GlyfsTer6 + **c.3291 + 3A > G**
#5 [30]	30	38	AR	HSP + dysarthria	Mild CA	POLR3A-related leukodystrophy NM_007055	**c.1909 + 22G > A +** c.3201_3202delGC/p.R1069fs*2
#6	35	57	AD	Spastic ataxia	CA	*PGN*/SPG 7 NM_003119.3	**c.1013G > T/p.G338V**
#7	40	48	SP	Progressive spastic ataxia (predominant pyramidal signs)	Mild hyperintense pyramidal tracts; spinal cord atrophy	*GJC2*/SPG44 (NM_020435)	c.219_220delCC (p.L74fs*33) + c.254T > C/(p.V85A)
#8	20	32	AR	Complex HSP + glaucoma	OPCA + medulla oblongata atrophy	*SYNE1*/SCAR8 NM_182961	c.15049C > T/p.Q5017* **(Homozygous)**
#9	13	56	AR	Severe spastic ataxia	normal	*TTPA*/AVED NM_000370D	**c.553-1G > T** **(Homozygous)**
#10 [31,37]	41	54	SP	Progressive spastic ataxia + psychosis; cognitive decline	Cortical atrophy; CA. Posterior leukoencephal	*TUBB4A* NM_006087	**c.545C > G/p.P182R** **(de novo)**
#11 [32]	30	42	SP	Gait disorder, axial and limbs dystonic tremor	CA	*PRKCG*/SCA14 NM_002739.4; NM_001316329.1	**c.380A > C/p.Q127P** **(de novo)**
#12 [36]	44	48	AD	Mild cerebellar ataxia and dysarthria	Severe CA	*STUB1*/SCA48 NM_001005920.2	c.673C > T/p.Arg225*
#13 [36]	51	54	SP	Cerebellar ataxia and cognitive decline	Severe CA	*STUB1*/SCA48 NM_001005920.2	**c.721C > T/p.Arg241Trp**
#14 [38]	38	46	AD	Gait ataxia + cognitive impairment	CA	TMEM240/SCA21 NM_001114748.2	c.509C > T/p.P170L [36]
#15 [39]	60	70	SP	Pure AC	CA	*KIF1B*/CMT2A1 NM_015074.3	**c.3845C > G/pA1282G**
#16	16	62	SP	Ataxic gait and dysarthria	CA	*PRKCG*/SCA14 NM_002739.4; NM_001316329.1	**c.1928T > G/p.F643C**

**Table 3:** Diagnostic yield comparison between results of this study and recent literature works on HSP and CA cohorts through NGS techniques. References numbers are in brackets. Abbrevations: HSP, Hereditary Spastic Paraplegia; CA, Cerebellar Ataxias; TGP, Targeted resequencing panels; TES, Targeted exome sequencing; WES, whole-exome sequencing.

References	Subjects	Diagnostic Rate HSP	Diagnostic Rate CA
Our data TGP	32	50%	29.2%
D’Amore et al. 2018 [30] TGP	239	29%	
Nemeth et al. 2013 [41] TGP	50		18%
Coutelier et al. 2018 [43] TES	319		28.5%
Lu et al. 2018 [44] TGP	55	61.8%	
Burguez et al. 2017 [8] TGP	29	48.3%	
Lynch et al. 2016 [45] TES + TGP	40	52.5%	
Kara et al. 2016 [46] TES + WES	97	49%	
Schule et al. 2016 [11] TGP + WES	608	46%	
Fogel et al. 2014 [28] TES	76		21%

## Data Availability

The data that support the findings of this study are available from the corresponding author, G.S., upon reasonable request.

## Supplementary Materials

The following are available online at https://www.mdpi.com/2076-3425/11/2/246/s1, Supplementary File: Description of cases diagnosed by Next-Generation Sequencing.
