# Supplementary material for: Application of a Clinical Workflow May Lead to Increased Diagnostic Precision in Hereditary Spastic Paraplegias and Cerebellar Ataxias: A Single Center Experience
Source: Brain Sci. 2021 Feb 16;11(2):246. doi: 10.3390/brainsci11020246 (PMC7919782; doi:10.3390/brainsci11020246)
Supplement: Supplementary file 1 [file brainsci-11-00246-s001.pdf]

Supplementary file

Description of cases diagnosed by Next-Generation Sequencing.

Pt #9, is a puzzling case: she is a 57 year-old Italian woman, born from non-consanguineous, healthy, Italian parents, referred to us for follow-up with a clinical diagnosis of Friedreich ataxia received even before molecular testing was available. Her clinical presentation was suggestive for a diagnosis of FRDA, as she suffered from a juvenile-onset progressive spastic-ataxia with dysarthria and sensory neuropathy. Neuroimaging showed no signs of cerebellar atrophy nor other significant pathological changes. Molecular tests performed in our center and repeated elsewhere only detected a heterozygous GAA expanded allele, and neither deletion/duplications nor punctuate mutations in the FXN gene. In addition, we observed qPCR levels of serum frataxin comparable to those we usually encounter in carrier relatives in FRDA families. NGS studies revealed a novel homozygous, likely pathogenic variant in the alpha tocopherol transfer protein (TTPA) gene causing AVED. Family segregation studies documented heterozygosity for this variant in both her parents and in her healthy brother. Although she was already severely affected, being wheelchair bound, starting of high doses vitamin E supplementation produced some improvement in both her upper limb coordination and swallowing function.

An additional HSP patient (pt #10) deserves mentioning. This male manifested psychiatric symptoms around the age of 20 years, featuring an obsessive-compulsive disorder; about 28 years later, he was sent to our evaluation for a subacute spastic-ataxia, associated with vitamin B12 deficiency. His symptoms were indeed partially responsive to vitamin B12 supplementation. During a long-lasting follow up, however, despite correct vitamin replacement, we observed a slowly progressive worsening of the spastic ataxia and the development of a cognitive decline. This prompted us to perform a further diagnostic assessment in this patient. Brain MRI initially documented mild cortical and cerebellar atrophy, then a follow-up study also revealed diffuse T2-hyperintensity of the cerebral hemispheric deep and subcortical white matter, predominantly of the parietal lobe, consistent with incomplete myelination, a TCC and mild putaminal atrophy. Further MRI studies showed permanent hypomyelination and some progression of the brain atrophy. His neurological status further worsened and the patient recently passed away at age of 49 years because of ab ingestis pneumonia. He harboured a novel, likely pathogenic heterozygous missense in TUBB4A arising de novo, as seen in previous reports documenting variants in this gene [37]. Interestingly, changes affecting the same residue (i.e., Proline 182) have already been described in association with the classic form of H-ABC [31].

Pt #14 is an Egyptian patient with adult-onset autosomal dominant ataxia associated with progressive cognitive impairment and cerebellar atrophy at brain MRI, in whom we detected an already described nonsense heterozygous pathogenic variant in the SCA21/TMEM240 gene [39] segregating with the disease.

Patient #7 is a 51 year-old woman affected by an adult onset, relatively rapidly evolving spastic paraplegia (wheelchair bound about ten years after the initial onset of symptoms). MRI studies showed mildly increased T2 signal of the cortico-spinal tracts, internal capsule and periventricular white matter and spinal cord atrophy). Family studies documented segregation with the phenotype of two variants (c.219_220delCC and c.254T>C) in the GJC2 gene.

Regarding VUS, a heterozygous variant in SPG7 (c.1013G>T) was detected in a proband (pt# 6) of a family affected by a slowly progressive spastic ataxia with a variable adult age at onset (range 20-50 years), associated with cerebellar atrophy at brain MRI. Disease transmission could be compatible with AD as well as a non-mendelian maternal inheritance. Extensive mtDNA studies ruled out major rearrangements or pathogenic point mutations in mtDNA, and direct sequencing of the AFG3LA2/ SCA 28 gene was also negative.

NGS studies documented another VUS in a distinct HSP-related gene, ATP2B4, encoding the plasma membrane calcium ATPase, that was also predicted as damaging/likely damaging by bioinformatics analysis and with very low frequency (0.00004) in the gnomAD database. Yet, only the VUS in SPG7 segregated with the disease, as it was detected in DNA samples from three affected family relatives while it was absent in the DNA from her healthy brother.

The VUS detected in pt #15 (c.3845C>G in the KIF1B gene) is predicted as possibly pathogenic by bioinformatics analysis. Family segregation studies could not be performed in this case. Although his phenotype would not match with that reported in association with KIF1B, this gene encodes a monomeric

motor for anterograde transport of mitochondria, and the only pathogenic heterozygous loss of function variant so far described has been linked to a rare AD Charcot Marie Tooth neuropathy (CMT2A1)

Finally, a VUS in PRKCG was detected in Pt #16, manifesting with a sporadic pure cerebellar ataxia with juvenile onset and very slowly progression.
